# Proangiogenic Hypoxia-Mimicking Agents Attenuate Osteogenic Potential of Adipose Stem/Stromal Cells

**DOI:** 10.1007/s13770-020-00259-3

**Published:** 2020-05-24

**Authors:** Ahmed G. Abu-Shahba, Arjen Gebraad, Sippy Kaur, Riku O. Paananen, Hilkka Peltoniemi, Riitta Seppänen-Kaijansinkko, Bettina Mannerström

**Affiliations:** 1 Department of Oral and Maxillofacial Diseases, University of Helsinki and Helsinki University Hospital, PO Box 63, 00014 Helsinki, Finland; 2 Department of Oral and Maxillofacial Surgery, Faculty of Dentistry, Tanta University, El-Gaish, Tanta Qism 2, Tanta, Gharbia Governorate Egypt; 3 Adult Stem Cell Group, Faculty of Medicine and Health Technology, Tampere University, Kalevantie 4, 33100 Tampere, Finland; 4 Helsinki Eye Lab, Ophthalmology, University of Helsinki and Helsinki University Hospital, Yliopistonkatu 4, 00100 Helsinki, Finland; 5 Laser Tilkka Ltd, Mannerheimintie 164, 2. krs, Helsinki, 00300 Finland

**Keywords:** Dimethyloxalylglycine, Baicalein, Hypoxia-inducible factor-1alpha, Adipose tissue-derived mesenchymal stem/stromal cells, Osteogenesis

## Abstract

**BACKGROUND:**

Insufficient vascularization hampers bone tissue engineering strategies for reconstructing large bone defects. Delivery of prolyl-hydroxylase inhibitors (PHIs) is an interesting approach to upregulate vascular endothelial growth factor (VEGF) by mimicking hypoxic stabilization of hypoxia-inducible factor-1alpha (HIF-1α). This study assessed two PHIs: dimethyloxalylglycine (DMOG) and baicalein for their effects on human adipose tissue-derived mesenchymal stem/stromal cells (AT-MSCs).

**METHODS:**

Isolated AT-MSCs were characterized and treated with PHIs to assess the cellular proliferation response. Immunostaining and western-blots served to verify the HIF-1α stabilization response. The optimized concentrations for long-term treatment were tested for their effects on the cell cycle, apoptosis, cytokine secretion, and osteogenic differentiation of AT-MSCs. Gene expression levels were evaluated for alkaline phosphatase (*ALPL*), bone morphogenetic protein 2 (*BMP2*), runt-related transcription factor 2 (*RUNX2*), vascular endothelial growth factor A (*VEGFA*), secreted phosphoprotein 1 (*SPP1*), and collagen type I alpha 1 (*COL1A1*). In addition, stemness-related genes Kruppel-like factor 4 (*KLF4*), Nanog homeobox (*NANOG*), and octamer-binding transcription factor 4 (*OCT4*) were assessed.

**RESULTS:**

PHIs stabilized HIF-1α in a dose-dependent manner and showed evident dose- and time dependent antiproliferative effects. With doses maintaining proliferation, DMOG and baicalein diminished the effect of osteogenic induction on the expression of *RUNX2*, *ALPL*, *and COL1A1*, and suppressed the formation of mineralized matrix. Suppressed osteogenic response of AT-MSCs was accompanied by an upregulation of stemness-related genes.

**CONCLUSION:**

PHIs significantly reduced the osteogenic differentiation of AT-MSCs and rather upregulated stemness-related genes. PHIs proangiogenic potential should be weighed against their longterm direct inhibitory effects on the osteogenic differentiation of AT-MSCs.

**Electronic supplementary material:**

The online version of this article (10.1007/s13770-020-00259-3) contains supplementary material, which is available to authorized users.

## Introduction

Large maxillofacial bone defects are a crippling problem from functional, psychological, and socioeconomic perspectives. Bone tissue engineering is a promising alternative to replace autografts and allografts in orthopedics and craniomaxillofacial surgery. It has developed rapidly owing to advances in cell biology and material science research [1–4].

Adipose tissue has gained much interest as a clinically appealing source for mesenchymal stem/stromal cells due to its abundance and accessibility with low donor site morbidity. Adipose tissue-derived mesenchymal stem/stromal cells (AT-MSCs) possess a great potential for bone tissue engineering and regenerative medicine strategies [5]. Such potential is suggested to be mediated through their immunomodulatory effects, antiapoptotic effects in osteoblastic lineage cells, recruitment of host stem/progenitor cells, pro-osteogenic effects, and stimulation of angiogenesis [6]. The proangiogenic role of mesenchymal stem/stromal cells, which significantly contributes to their regenerative potential, is mainly exerted via their paracrine effects which act in a vascular endothelial growth factor (VEGF)-dependent manner [6–8].

Despite the major advances in the field of tissue engineering, clinical applications remain restricted mainly to thin or avascular tissues, such as skin, cartilage, and cornea. Larger tissue constructs like bone require a vasculature that provides the cells with oxygen and nutrients for long-term survival post-implantation [9–13]. Previous preclinical and clinical trials have focused on enhancing vascularization through the local delivery of VEGF. However, the short half-life of VEGF reduces its efficacy, and increasing the dose to super-physiological levels increases its systemic side-effects and risk of complications [14].

The induction of a therapeutic hypoxic response is an interesting alternative with clinically relevant potential. Cells react to low oxygen levels with a hypoxic response, of which the hypoxia-inducible transcription factor (HIF-1) is a key regulator [15]. HIF-1 binds hypoxic response element (HRE)-driven promoters on several genes, including *VEGF*, *glucose transporter*-*1*, *and erythropoietin* [15, 16]. HIF-1 is a heterodimeric complex composed of HIF-1α and HIF-1β with HIF-1α being the oxygen-regulated subunit [17]. HIF-1α is highly unstable in normoxic conditions, as it undergoes proteosomal degradation. The process is initiated by oxygen-dependent hydroxylation of HIF-1α under the control of prolyl hydroxylases (PHD 1, 2, and 3) and an asparaginyl hydroxylase known as Factor Inhibiting HIF-1α (FIH). These hydroxylases require iron, oxygen, and 2-oxaloglutarate (2-OG) as cofactors for the hydroxylation process, which in turn leads to Von Hippel-Lindau protein (pVHL)-mediated ubiquitination and subsequent proteasomal degradation of HIF-1α [18].

Under physical hypoxic conditions, hydroxylase enzymes are inactive. Prolyl-hydroxylase inhibitors (PHIs) can mimic the hypoxic response in normoxic conditions by modulating HIF-1α degradation. Such a chemically-induced hypoxic response occurs in the presence of iron chelators such as deferoxamine, or in the presence of a 2-OG competitive inhibitors such as dimethyloxalylglycine (DMOG) [18]. Baicalein is an active flavonoid extracted from the root of the plant *Scutellaria baicalensis*, it has been reported to be a PHD2 inhibitor, which abrogates asparaginyl hydroxylation of HIF-1 [19].

From the perspective of bone tissue engineering, HIF-1α stabilization potentially aims at enhancing the proangiogenic effects of AT-MSCs. Chemical stabilization of HIF-1α by PHIs could have other effects on AT-MSCs behavior in the context of bone tissue regeneration. In this study, we aim to investigate DMOG and baicalein for their effects on HIF-1α stabilization, proliferation and osteogenic differentiation of AT-MSCs, as potential candidates for targeting angiogenesis-osteogenesis coupling in bone tissue engineering.

## Materials and methods

### Adipose tissue-derived mesenchymal stem/stromal cells isolation

Water-assisted liposuction-aspirates served as the source of AT-MSCs. The donors comprised seven females with an age range of 32–50 (average of 41 ± 7.4), and body mass index range of 22.7–31.2 (average of 26.4 ± 3.1). Informed consents were obtained under the ethical approval of the ethical committee of Helsinki and Uusimaa Hospital District for the use of adipose tissue in scientific research. Fresh liposuction-aspirates underwent a combination of enzymatic and mechanical treatment for the isolation of AT-MSCs as previously described [20, 21].

Cell cultures were maintained at 37 °C and 5% CO_2_ in a humidified incubator. The plastic-adherent AT-MSCs were expanded in maintenance medium (MM) with full medium change every 3 days, passaging at 1:3 split ratio when 85% confluent. Cells from passages 3-6 were used in all experiments. The MM consisted of Dulbecco’s modified Eagle’s medium/Ham’s Nutrient Mixture F-12 with 1% l-alanyl-l-glutamine (DMEM/F-12 1:1 GlutaMAX; ref. 31331-028, Gibco, Grand Island, NY, USA), 1% antibiotics (100 U/mL penicillin, 0.1 mg/mL streptomycin; ref. DE17-602E, Lonza), and 10% fetal bovine serum (FBS; South American, ref. 10270-106, Gibco).

### Characterization of AT-MSCs

Surface antigens of interest on the AT-MSCs were detected using a BD Accuri C6 flow cytometer (Becton–Dickinson, Franklin Lakes, NJ, USA) and allophycocyanin (APC)-conjugated monoclonal antibodies against CD14 (clone: M5E2), CD19 (clone: HIB19), CD34 (clone: 581), CD45RO (clone: UCHL1), CD54 (clone: HA58), CD73 (clone: AD2), CD90 (clone: 5E10), CD105 (clone: 266), and HLA-DR (clone: G46-6) (BD Pharmingen, Becton-Dickinson, Franklin Lakes, NJ, USA) [22]. We performed flow cytometric analysis of AT-MSCs expanded in MM up to passage 5 using 1 × 10^4^ events recorded per sample. Expression was considered positive when the level of fluorescence was greater than 99% of the corresponding unstained cell sample [23]. Multipotentiality of AT-MSCs was assessed by analysing their capacity to differentiate toward the adipogenic, osteogenic and chondrogenic lineages. The adipogenic differentiation involved culturing AT-MSCs for 3 weeks in adipogenic media (StemPro^®^ Adipogenesis Differentiation Kit, # A10070-01, Gibco). Osteogenic differentiation media (OM) consisted of MM supplemented with 50 µM l-ascorbic acid 2-phosphate, 10 mM β-glycerophosphate disodium salt hydrate, and 5 nM dexamethasone (all from Sigma-Aldrich). Chondrogenic differentiation was tested in high density spheroid culture conditions, it was induced by chondrogenic medium consisting of MM with reduced FBS to 1% and supplemented with 1% Insulin-Transferrin-Selenium-Ethanolamine (ITS-X, # 51500056, Gibco), 50 μg/mL l-ascorbic acid 2-phosphate (Sigma-Aldrich), 40 μg/mL l-proline (Sigma-Aldrich), 100 μg/mL sodium pyruvate (# 11360070, Gibco), 100 nM dexamethasone (Sigma-Aldrich), and 10 ng/mL of TGF-β1 (# 7754-BH-005, R&D system, Minneapolis, MN, USA).

### Assessing the short-term viability/cytotoxicity aspects of DMOG and baicalein

Both small-molecule drugs; DMOG (D3695, CAS: 89464-63-1, lot # 086M4731V) and baicalein (465119, CAS: 491-67-8, lot # MKBV1595V) were from Sigma-Aldrich (St. Louis, MO, USA). DMOG and baicalein were readily soluble in DMSO (MP Biomedicals, LLC, Illkirch Cedex, France). Cell Counting Kit-8 (CCK-8) (# CK04-11, Dojindo Molecular Technologies, Rockville, MD, Maryland, USA) was used according to the manufacturer instructions to assess the cytotoxicity of varying concentrations of DMOG and baicalein, control conditions received only DMSO in equal volumes. AT-MSCs were cultured on 96-well plates at 1.6 × 10^3^ cells/well in 100 µL of MM and allowed to attach for 24 h in a humidified incubator. Cells then received test conditions of MM with 100, 200, and 500 µM of DMOG, or 5, 10, 50 and 200 µM of baicalein. DMSO in MM (0 µM PHIs) served as a control condition and wells without cells were used as blanks. On treatment days 0, 1, and 2 each well received 10 μL of CCK-8 solution. The absorbance was measured after 3 h of culture at 450 nm using a microplate reader (PerkinElmer VICTOR™ X4 Multilabel Microplate Reader 2030, Turku, Finland).

### Assessing DMOG and baicalein effects on cellular HIF-1α levels

AT-MSCs were seeded in MM at a density of 2 × 10^4^ cells/well on sterile coverslip inserts in 24-well culture plates. After overnight attachment, AT-MSCs were cultured for 5 h in MM with 500 µM DMOG, or 185 µM baicalein. AT-MSCs cultured in MM with DMSO or 100 µM CoCl_2_·6H_2_O (Merck, Darmstadt, Germany. Art.2539) served as negative and positive controls, respectively. Cells were fixed in 4% paraformaldehyde for 10 min and rinsed 3 × 5 min in PBS. Fixed cells were permeabilized using 0.5% Triton X-100 for 20 min, followed by washing in PBS. After blocking in 10% normal donkey serum for 1 h at RT, cells were incubated overnight with primary mouse anti-human HIF-1α (# 610959, BD Biosciences, Franklin Lakes, NJ, USA) at a dilution of 1:50 in 0.5% normal donkey serum at 4 °C. Coverslips were rinsed, then incubated with both donkey anti-Mouse IgG secondary antibody (Alexa Fluor 568, # A10037, Life Technologies, Eugene, OR, USA) in 5 µg/ml dilution and CellTrace™ 1:1000 (CFSE Cell Proliferation Kit, # C34554, Life technologies) for 1 h at RT. Nuclei were stained in Hoechst 33342 (# B2261, Sigma-Aldrich) for 30 min in the dark, followed by washing and mounting on a glass slide with SlowFade^®^ mountant (# S36967, Thermo Fisher Scientific, Waltham, MA, USA). We used a Leica TCS SP8 confocal microscope (Leica Microsystems GmbH, Wetzlar, Germany) to image the cells.

### Western blotting for HIF-1α and VEGF

AT-MSCs seeded at a density of 3 × 10^5^ cells/well in 6-well culture plates were cultured for 5 h in MM with 500 µM DMOG or 185 µM baicalein. MM with DMSO or 100 µM CoCl_2_·6H_2_O (Merck, Art. 2539) served as negative and positive controls, respectively. AT-MSCs were lysed with 1 × cell lysis buffer (# 9803, Cell Signaling Technology). After measuring protein concentrations, by Pierce™ BCA Protein Assay Kit (# 23227, Thermo Fisher Scientific), samples containing 22 µg protein were denatured at 95 °C for 5 min in reducing Laemmli sample buffer, separated using Mini-PROTEAN^®^ TGX™ 12% gradient SDS-PAGE gel (Bio-Rad, Hercules, CA, USA) with BlueSTAR prestained protein marker (# MWP03, Nippon Genetics Europe GmbH) as a standard. Running conditions were 150 V for 60 min. Blotting involved semi-dry transfer of proteins on nitrocellulose membranes of 0.2 µm pore size (#162-0112, Bio-Rad), using 40 mA per gel for 60 min.

For normalizing the target signal, we utilized REVERT™ Total Protein Stain kit (# 926-11010, LI-COR, Lincoln, NE, USA) according to manufacturer’s instructions. Blocking and antibody incubations were performed in Odyssey blocking buffer (LI-COR) without and with 0.1% Tween-20, respectively. Primary antibodies of mouse anti-human HIF-1α (1:500, # 610959, BD Biosciences) and VEGF antibody (C-1) (1:200, # sc-7269, Santa Cruz, Dallas, TX, USA) were used for immunodetection. After primary antibody overnight incubation at + 4 °C, membranes were washed in TBS-T and probed with secondary IRDye^®^ 800CW Goat (LI-COR) at 1: 15,000 for 1 h at RT. Odyssey FC Imager (LI-COR) and Image Studio™ Software served to quantify the lane normalization factor and normalizing the immunodetected-signal.

### HIF-1α stabilization response for DMOG and baicalein doses

AT-MSCs were plated in black 96-well clear-bottom plates (# 6005182, Perkin-Elmer) at a cell-density of 1 × 10^4^ cells/well and allowed to adhere overnight. Cells were cultured for 5 and 72 h in MM with 312.5, 625, and 1563 µM of DMOG, or 16, 32, 162, and 624 µM of baicalein, or with equal volumes of DMSO. The HIF-1α stabilization was quantified using the In-Cell ELISA Near Infrared Detection Kit (# 62201, Thermo Fisher Scientific) following the manufacturer’s instructions. Cells were fixed in 4% methanol-free formaldehyde (# 28906, Thermo Scientific), followed by washing, permeabilization, and blocking steps. HIF-1α was probed using mouse anti-human HIF-1α (1:50, # 610959, BD Biosciences), and an antibody for housekeeping protein rabbit polyclonal beta actin (2 µg/mL, # PA5-16914, Thermo Fisher Scientific). After overnight incubation at + 4 °C, AT-MSCs were washed with 1 × wash buffer before incubation with species-specific near-infrared DyLight-conjugated secondary antibody mix for 1 h at RT. After washing steps, Odyssey FC Imager (LI-COR) served to scan the plates with excitation/emission maxima of 692/712 nm for DyLight 680 Dye and 777/794 nm for DyLight 800 Dye. Measured signals were analyzed with Image Studio Software (LI-COR).

### Assessing the long-term viability/cytotoxicity aspects of DMOG and baicalein

Viability/cytotoxicity responses over prolonged exposure to PHIs was assessed using CCK-8 assay and CyQUANT™ Cell Proliferation Assay Kit (# C7026, Invitrogen). AT-MSCs were seeded at a density of 1.6 × 10^3^ cells/well in 96-well plate and 100 µL of MM per well was added containing 100, 200, and 500 µM of DMOG, or 5, 10, 50, and 200 µM of baicalein. Cell viability was either assayed using the CCK-8 assay following manufacturer’s protocol at 0, 3, 6, 10, and 14 days of treatment, or cells were lysed in 0.1% Triton-X 100 (Sigma-Aldrich) after 0, 1, 3, 7, and 14 days of treatment. A freeze–thaw cycle assisted cell lysis prior to analysis. CyQUANT™ cell proliferation assay kit was used to estimate the total cellular DNA in cell lysates. Fluorescence was measured with VICTOR™ microplate reader at 480/520 nm excitation/emission maxima. Proliferation assays were used for estimating the optimized PHIs concentrations for long-term treatment of AT-MSCs.

### Cell cycle analysis and Annexin V/PI Flow cytometric assay

The optimized PHIs concentrations were 3.75 pmol/cell of DMOG and 0.25 pmol/cell for baicalein. These concentrations were assessed with regards to their effects on the cell cycle and apoptosis of AT-MSCs after 4 days and 14 days of treatment. For cell cycle analysis, harvested ethanol-fixed cells were stained with FxCycle™ PI/RNase Staining Solution (# F10797, Thermo Fisher Scientific) according to the manufacturer’s protocol, then underwent flow cytometry using BD Accuri C6 flow cytometer. For apoptosis assay, harvested cells were washed and stained using Dead Cell Apoptosis Kit with Annexin V Alexa Fluor™ 488 and Propidium Iodide (PI) (# V13241, Thermo Fisher Scientific) according to the manufacturer’s protocol, followed by flow cytometric analysis using BD Accuri C6.

### Human cytokine antibody assay for AT-MSCs conditioned media

AT-MSCs were seeded in 48-well plates at a density of 1 × 10^4^ cells/well. The AT-MSCs were cultured in 300 µL/well of MM or OM supplemented with 3.75 pmol/cell of DMOG, 0.25 pmol/cell baicalein, or equal volumes of DMSO. After 14 days of treatment, cells were cultured in serum-free medium for 24 h. Conditioned medium was collected and centrifuged (1000*g* for 10 min, + 4 °C). The supernatant was stored in − 80 °C until further analysis. Levels of secreted cytokines of interest were measured using human cytokine antibody array membranes (# ab133998, Abcam, Cambridge, UK) following the manufacturer’s protocol. Briefly, membranes were incubated in blocking buffer (30 min, RT). Equal amounts of proteins were pooled from three independent experiments to achieve the same protein concentration in all samples. Samples were incubated overnight on membranes at +4 °C under gentle shaking. Thorough washing steps of membranes preceded incubation at with biotin-conjugated anticytokines. Membranes were washed thoroughly and incubated with HRP-conjugated streptavidin overnight at +4 °C. After washing, the membranes were blot-dried and incubated with the detection buffer for 2 min at RT and were then imaged using ChemiDoc XRS Imaging System (Bio-Rad). ImageJ software (National Institutes of Health) was used to quantify the intensity of individual dots by densitometric analysis. MM with DMSO served as reference, and normalized signal density of each dot was then calculated. Due to the low number of negative controls; 2 per membrane, the background error was estimated by taking the difference between the average of negative controls and the lowest value on the assay. Only cytokines detected above experimental error in at least one sample were included in the analysis.

### Biochemical analyses for osteogenic response

The osteogenic potential of AT-MSCs was assessed under the concentrations and experimental setting described in Sect. 2.9 using alkaline phosphatase (ALP) assay, hydroxyproline assay, and Alizarin Red S stain (ARS) after 14 days of treatment. In order to assess the effect of collagen-I on mineralization response of AT-MSCs by ARS, culture was repeated on plates coated with Rat tail collagen-I (# 354236, CORNING™, Corning, NY, USA) according to manufacturer’s instructions. AT-MSCs were lysed using 0.1% triton-x-100 and freezing at − 80 °C. Alkaline phosphatase activity was measured by mixing the cell lysate with *p*-nitrophenyl phosphate disodium (# P5744, Sigma-Aldrich) and 2-amino-2-methyl-1-propanol (# A9226, Sigma-Aldrich). The amount of produced *p*-nitrophenol was measured in microplate reader at 405 nm. Hydroxyproline assay Kit (# MAK008, Sigma-Aldrich) was employed according to the manufacturer’s protocol to determine the hydroxyproline concentration in cell lysates, correlating with the collagen content. Briefly, after hydrolyzing cell lysates in 6 N hydrochloric acid at 120 °C for 3 h, oxidized hydroxyproline was allowed to react with 4-(dimethylamino) benzaldehyde for 90 min at 60 °C. Absorbance of the colorimetric product was measured at 550 nm in the microplate reader. To normalize the alkaline phosphate activity and total collagen content for cell number, the amount of DNA in the cell culture lysates was quantified using CyQUANT™ cell proliferation assay.

For Alizarin Red S Staining (ARS), AT-MSCs were fixed in ice-cold 70% ethanol for 1 h at RT, followed by rinsing twice with ddH2O. Cells were stained with 2% ARS solution (# A5533-25G, Sigma-Aldrich) for 30 min at RT, followed by thorough washing for 5 × 5 min in fresh ddH2O under shaking. Bound ARS was extracted using 100 mM N-cetylpyridinium chloride monohydrate (Merck) in ddH_2_O for 2 h in 37 °C followed by measuring the eluted stain at 550 nm in the microplate reader. To normalize the ARS/cell, fixed cells were stained by 2 mM Janus Green B Stain (# 201677-25G, Sigma-Aldrich) for 5 min at RT. After washing for 5 × 5 min in fresh ddH_2_O, 0.1 mL 0.5 M HCL was added per well and left for 10 min to elute the stain. This was followed by shaking the plate for 10 s and measuring the absorbance at 550 nm in the microplate reader.

### Gene expression analyses by quantitative PCR (qRT-PCR)

We analyzed gene expression by AT-MSCs at 7 days of treatment. Total RNA was isolated using the miRCURY™ RNA Isolation Kit (Exiqon A/S) according to the manufacturer’s instructions. Reverse transcription into cDNA was done using a SuperScript™ IV VILO™ reaction mixture (# 11766050, Thermo Fisher Scientific). PCR reactions were conducted on a QuantStudio™ 5 Real-Time PCR System (Thermo Fisher Scientific) using TaqMan^®^ assays (Thermo Fisher Scientific) for the following genes: alkaline phosphatase, liver/bone/kidney (*ALPL*, assay ID Hs01029144_m1), bone morphogenetic protein 2 (*BMP2*, assay ID Hs00154192_m1), runt-related transcription factor 2 (*RUNX2*, assay ID Hs01047973_m1), vascular endothelial growth factor A (*VEGFA*, assay ID Hs00900055_m1), secreted phosphoprotein 1 (*SPP1*, assay ID Hs00959010_m1), collagen type I alpha 1 (*COL1A1*, assay ID Hs00164004_m1), and ribosomal protein lateral stalk subunit P0 (*RPLP0*, assay ID Hs99999902_m1) as a house keeping gene for normalization.

Stemness-related genes Kruppel-like factor 4 (*KLF4*), Nanog homeobox (*NANOG*), and octamer-binding transcription factor 4 (*OCT4*) were assessed using primers at 2 μM concentration (Table 1), and 5 × HOT FIREPol EvaGreen qPCR Mix Plus (no ROX) (# 08-25-00001, Solis BioDyne, Tartu, Estonia) in a final volume of 20 µL and run in Rotor-Gene Q (Qiagen, Hilden, Germany). Cyclophilin G (*CycloG*) served as an endogenous housekeeping control gene for stemness-related genes. Data were analyzed using the 2^−ΔΔCt^ method to quantify relative gene expression [24].

**Table 1:** Primers used for assessing stemness-related genes

Genes	Sequence of 5′-primer (F)	Sequence of 3′-primer (R)	Size (bp)	Origin
*KLF4*	5′-CCGCTCCATTACCAAG-3′	5′-CACGATCGTCTTCCCCTCTT-3′	80	hum (NM_004235.4)
*NANOG*	5′-CTCAGCCTCCAGCAGATGC-3′	5′-TAGATTTCATTCTCTGGTTCTGG-3′	94	hum (NM_024865.2)
*OCT4*	5′-TTGGGCTCGAGAAGGATGTG-3′	5′-TCCTCTCGTTGTGCATAGTCG-3′	91	hum (NM_002701)
*CycloG*	5′-TCTTGTCAATGGCCAACAGAG-3′	5′-GCCCATCTAAATGAGGAGTTG-3′	84	hum (NM_004792)

### Statistical analysis

Statistical analyses were performed using arithmetic mean of at least three technical replicates from three AT-MSCs donors (biological replicates) by Origin 2018 (OriginLab, Northampton, MA, USA). Analysis of variance (ANOVA) was employed; either one-way or two-way depending on independent variables. Bonferroni-corrected post hoc means comparison tests were performed to analyze specific sample pairs for significant differences. The results were considered significant when *p* < 0.05.

## Results

### AT-MSCs characterization

**Fig. 1:**
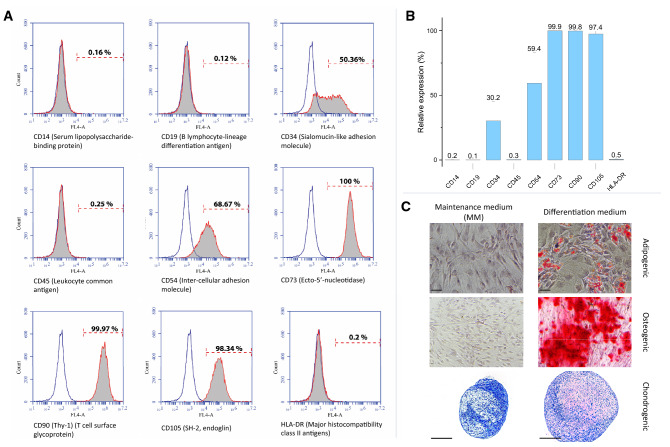
AT-MSCs characterization; **A** Surface markers’ expression of undifferentiated AT-MSCs cultured in MM as analyzed by flow cytometry. Empty histograms show signal from unstained control cells, while grey-filled histograms show signal of cells stained with antibody against the surface proteins. **B** A column graph shows mean and SD for surface marker expression levels (%) of undifferentiated AT-MSCs (n = 7). **C** Photomicrographs show multipotentiality of tested AT-MSCs when induced for 3 weeks towards adipogenesis (Oil Red O staining; upper row), osteogenesis (Alizarin Red S staining; middle row), and chondrogenesis (Toluidine blue staining; lower row). Maintenance medium (MM) was used in parallel as a negative control for differentiation conditions. Scale bar = 200 μm

AT-MSCs showed the characteristics defined by the International Federation for Adipose Therapeutics (IFATS) and Science and the International Society for Cellular Therapy (ISCT) [22, 25]. The plastic-adherent AT-MSCs had fibroblast-like morphology, expressed surface markers CD73, CD90 and CD105, while lacking the expression of hematopoietic markers CD14, CD19, CD45 and HLA-DR (Fig. 1A). The cells showed a moderate expression of CD34 and evident donor variability in the expression of CD34 and CD54 (Fig. 1B).

AT-MSCs showed the capacity for adipogenic differentiation with gradual accumulation of lipid intracellular droplets, detected by Oil Red O stain (Fig. 1C). Osteogenic differentiation under described induction condition was successful (Fig. 1C and supplementary Fig. 3). The chondrogenic differentiation for AT-MSCs spheroids was assessed by Toluidine blue staining which showed a purple metachromatic stain confirming the presence of extracellular glycosaminoglycans when cells were cultured in chondrogenic induction medium (Fig. 1C).

### Short-term viability/cytotoxicity aspects of DMOG and baicalein

**Fig. 2:**
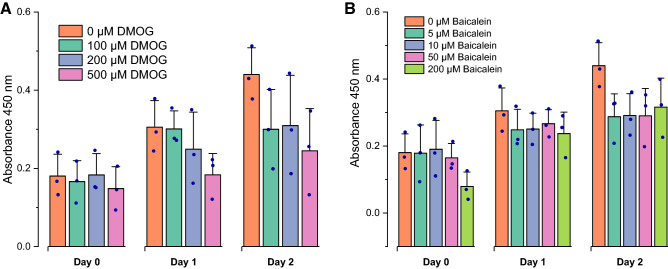
Short-term viability of AT-MSCs in presence of DMOG (**A**) and baicalein (**B**); CCK-8 assay results for AT-MSCs cultured for 2 days in presence of various concentrations of DMOG (**A**) and baicalein (**B**). 0 µM is MM + DMSO. Column graphs represent mean and SD for three independent biological replicates (dots)

AT-MSCs viability declined with 500 µM of DMOG on the second day of treatment as compared to control condition but the difference was not significant (Fig. 2A). Although cellular viability relatively decreased with baicalein treatment as compared to control, the difference was non-significant (Fig. 2B). These results reveal that concentrations up to 500 µM DMOG and 200 µM baicalein are suitable for short-term treatment of AT-MSCs.

### DMOG and baicalein stabilize HIF-1α in AT-MSCs

**Fig. 3:**
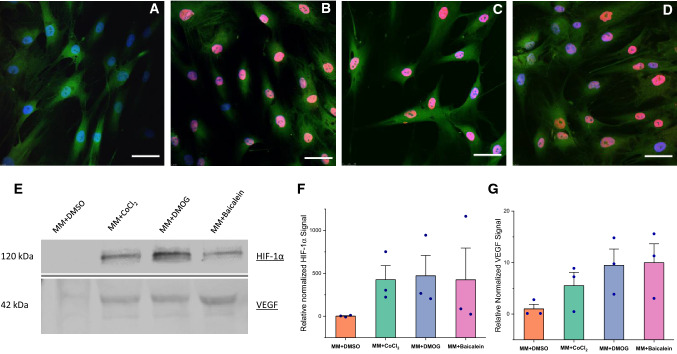
DMOG and baicalein stabilize HIF-1α in AT-MSCs; confocal microscopy images for HIF-1α immunostained samples of AT-MSCs treated with MM + DMSO (**A**) as a negative control, MM + CoCl_2_.6H_2_O as a positive control (**B**), MM + DMOG (**C**), and MM + Baicalein (**D**). Stabilized HIF-1α translocated into the nuclei in all conditions except negative control. Western blotting of HIF-1α and VEGF (**E**), and band density analysis normalized to total protein stain (REVERT™) (**F**, **G**). Both drugs as well as positive control stabilized HIF-1α and increased VEGF levels. Column graph represents mean and SE for three biological replicates (dots); Scale bar 50 µm

**Fig. 4:**
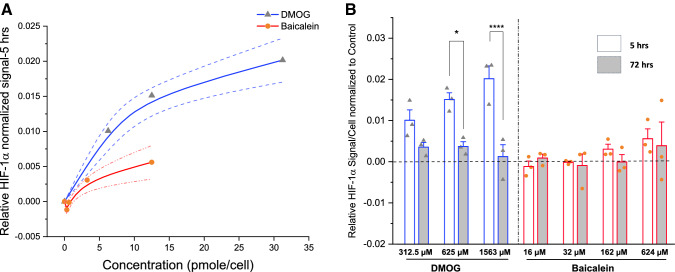
HIF-1α stabilization response in different doses overtime; In-Cell ELISA assay results showing dose-dependent stabilization of HIF-1α by DMOG and baicalein at 5 h. **A** After 72 h, HIF-1α levels declined significantly with DMOG (**B**). The line graph in **A** shows three donors’ mean (line) ± SE (dotted lines) response for tested drug concentrations expressed in pM/cell. Column graph in **B** shows observed mean and SE for three independent biological replicates (Symbols; Δ and Ο), horizontal dashed reference line denotes relative signal of control condition (MM + DMSO), *p ≤ 0.05; ****p ≤ 0.0001

Immunostaining revealed the localization of HIF-1α in the nuclei of cells treated with DMOG and baicalein. This confirmed the ability of both tested drugs to stabilize cellular HIF-1α, which mainly translocated into the nucleus (Fig. 3A–D). Western blotting also confirmed stabilized HIF-1α and VEGF expression in treated samples as well as in positive controls, although variability in donor response was evident (Fig. 3E–G). Both tested drugs stabilized HIF-1α in a dose dependent manner, DMOG showed higher potency to stabilize HIF-1α, the response to DMOG rapidly declined, whereas the lower HIF-1α stabilization effect of baicalein did not show significant decrease overtime (Fig. 4).

### Long-term viability/cytotoxicity aspects of DMOG and baicalein

**Fig. 5:**
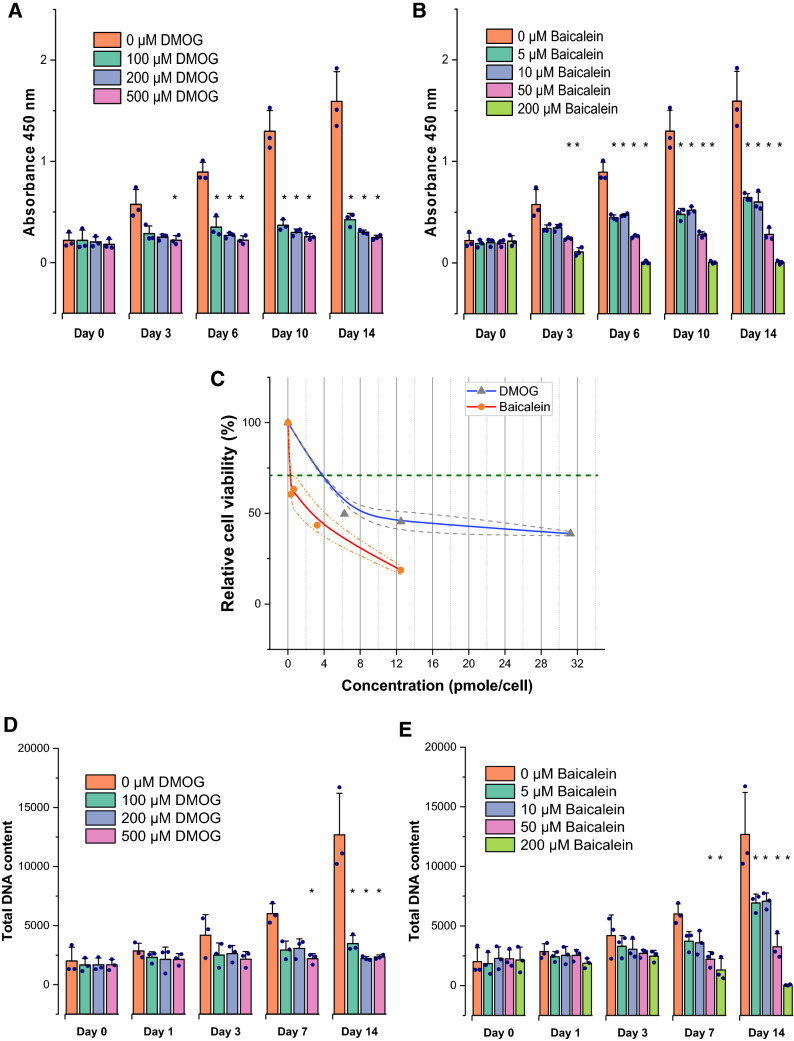
Long-term viability of AT-MSCs in presence of DMOG and baicalein; results of CCK-8 assay for cellular proliferation and metabolic activity (**A**, **B**) and CyQUANT assay for DNA content (**D**, **E**) up to 14 days of treatment. Significant deterioration of cellular proliferation and metabolic activity was detected prior to the reduction in DNA content. Earliest significant cytostatic/cytotoxic effects were detected with CCK-8 on the third day, these results were employed to develop a cell viability-concentration curve (**C**) in which cell viability is expressed as relative to the control. The line graph in **C** shows mean (line) ± SE (dotted lines) relative viability at tested drug concentrations; horizontal dashed reference line denotes the aimed 70% viability level; corresponding drug concentrations were employed in subsequent analyses. Column graphs show observed mean and SD for three independent biological replicates (dots), *p ≤ 0.05 compared to 0 µM (MM + DMSO) control

CCK-8 results showed a significant effect of DMOG on cell viability for drug concentration (*F* (3, 40) = 148.9, *p* < 0.0001), for the duration of treatment (*F* (4, 40) = 33.9, *p* = 2.3 × 10^−12^), and a significant interaction (*F* (12, 40) = 17.9, *p* = 1.9 × 10^−12^). The cell viability significantly deteriorated with 500 µm DMOG on the third day of treatment (Fig. 5A). Baicalein showed a significant main effect for drug concentration (*F* (4, 50) = 189.5, *p* < 0.0001), for the duration of treatment (*F* (4, 50) = 50.9, *p* < 0.0001), and a significant interaction (*F* (16, 50) = 21.8, *p* < 0.0001). Cytotoxicity of baicalein started to be significant on the third day with 50 µM and 200 µM baicalein (Fig. 5B). A dose–response curve, for the CCK-8 results on third day, was used to estimate the optimized concentrations for long-term treatment of AT-MSCs corresponding to a viability percentage of 70% (Fig. 5C). We found it more reproducible to express concentrations in moles per cell [26]. Optimized concentrations were 3.75 pmol/cell of DMOG and 0.25 pmol/cell of baicalein (Fig. 5C). Total DNA content measurements were in line with CCK-8 results; however, significant cytotoxicity was evident only after 3 days (Fig. 5D, E). These findings suggest an earlier effect on the metabolic activity of the cells, which was evident at the third day.

**Fig. 6:**
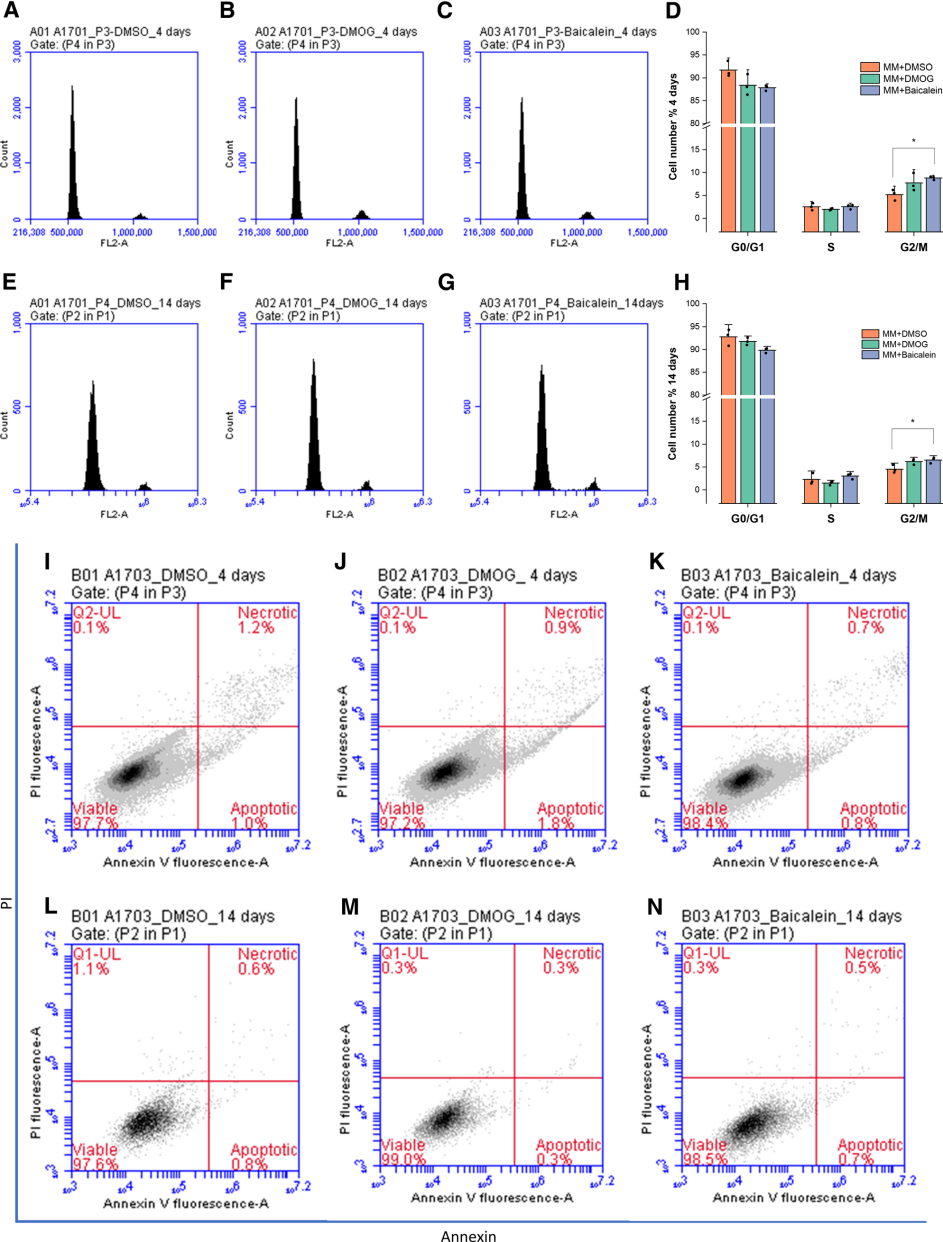
Cell cycle analysis and Annexin V/PI flow cytometric assay results; cell cycle representative histograms of the gated cells after 4 days (**A**–**C**) and 14 days of treatment with PHIs (**E**–**G**). Quantitative analysis of distribution of the cells in each phase was performed from at least 10,000 cells per sample after 4 days (**D**) and 14 days of treatment (**H**). Annexin V/PI flow cytometric analysis for AT-MSCs treated with PHIs for 4 days (**J**, **K**) and 14 days (**M**, **N**), as compared to control cells treated with DMSO (**I**, **L**). The results did not show a significant cellular apoptosis or necrosis at tested concentrations for both durations as compared to DMSO control. Column graphs show observed mean and SD for three independent biological replicates (dots). *p < 0.05 versus control

Cell cycle analysis of AT-MSCs treated with optimized concentrations of PHIs revealed, in both treatment durations, that PHIs moderately increased the percentage of cells in G2/M phase at the expense of G0/G1 phase (Fig. 6A–H). Annexin V/PI flow cytometric analysis results, did not show a significant cellular apoptosis or necrosis at tested concentrations for both durations as compared to DMSO control (Fig. 6I–N).

### DMOG and baicalein attenuated the osteogenic potential of AT-MSCs

**Fig. 7:**
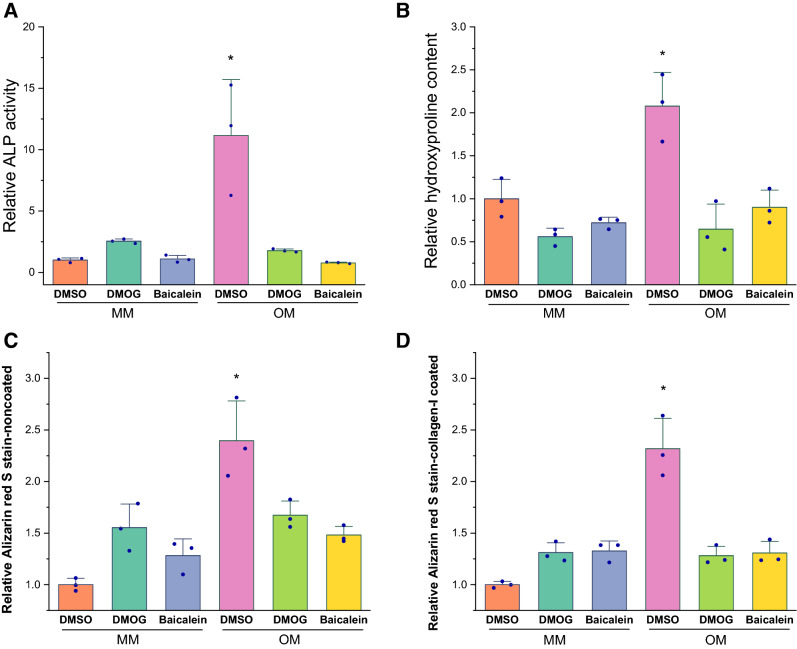
DMOG and baicalein attenuated AT-MSCs osteogenic induction; quantified normalized ALP activity (**A**), collagen content (**B**), and mineralization content (**C**, **D**). Both drugs significantly reduced the osteogenic response of cells to levels comparable to their non-osteogenic induction counterparts (**A**–**C**), mineralization ARS assay showed similar finding even with collagen-I precoated plates (**D**). Column graphs show observed mean and SD for three independent biological replicates (dots), *p < 0.05 compared to other conditions

AT-MSCs showed an osteogenic response at 14 days of treatment exclusively with OM + DMSO, whereas tested PHIs attenuated osteogenic induction (Fig. 7). One-way analysis of variance for ALP assay results showed that average relative ALP activity for OM + DMSO was significantly higher (*p* < 0.001) than all other test conditions, adding PHIs to OM reduced ALP activity (Fig. 7A). Relative collagen content, as estimated by hydroxyproline assays, showed a significantly higher collagen content in OM + DMSO than in the other test conditions (*p* < 0.05) (Fig. 7B). The ARS assay results also showed that average relative mineralization for OM + DMSO was significantly higher (*p* < 0.001) than all other test conditions (Fig. 7C), whereas adding PHIs to OM significantly reduced the relative mineralization. Collagen coating of the plates did not enhance mineralization (Fig. 7D).

### Cytokine analysis for AT-MSCs conditioned media

**Fig. 8:**
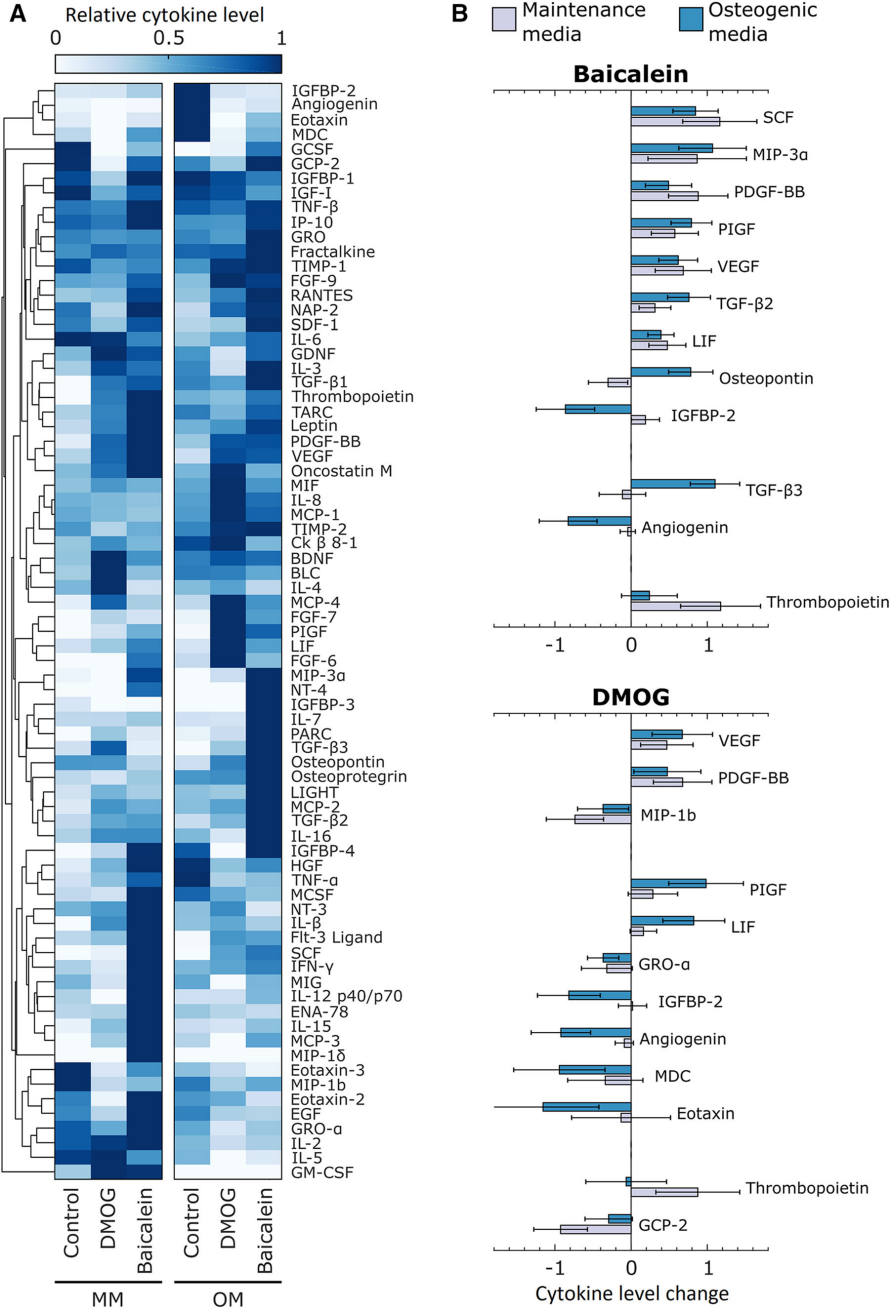
Cytokine array analysis results; showing unsupervised clustering analysis of all the detected cytokines (**A**) and relative change in concentration of 12 most significant cytokines induced by baicalein and DMOG (**B**). In **B**, cytokines are grouped in three groups: Top = change of concentration levels in both maintenance media (MM) and osteogenic media (OM), middle = change of concentration levels only in OM, bottom = change of concentration levels only in MM

Out of the 80 cytokines included in the cytokine array 75 were detected in at least one sample. Unsupervised clustering analysis (Fig. 8A) showed that DMOG and baicalein had distinctive effects on cytokine levels, and their effects differed in MM and OM. Similar effects were observed for baicalein and DMOG, especially in angiogenesis-related cytokines (Fig. 8B). Both DMOG and baicalein increased VEGF and platelet-derived growth factor-BB (PDGF-BB) levels in OM and MM media. However, DMOG decreased the concentration of several CC chemokines, such as macrophage inflammatory protein-1b (MIP-1b), macrophage-derived chemokine (MDC), eotaxin, and CXC chemokines like growth regulated α protein (GRO-α) and granulocyte chemotactic protein 2 (GCP-2). Baicalein instead increased levels of stem cell factor (SCF), macrophage inflammatory protein-3 (MIP-3α), and transforming growth factor-beta 2 (TGF-β2) in both MM and OM, and increased the concentration of transforming growth factor-beta 3 (TGF-β3) and osteopontin in OM.

### Quantitative PCR (qRT-PCR) results

**Fig. 9:**
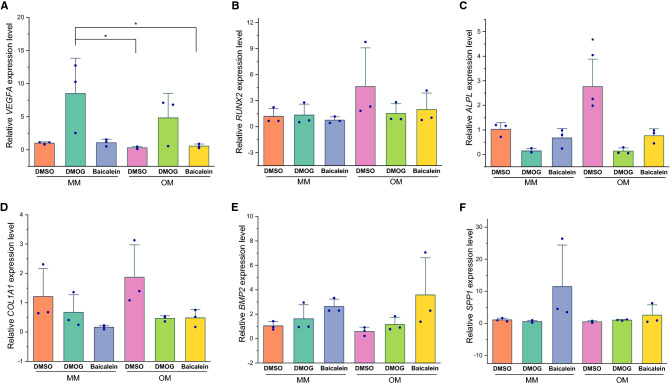
Expression of *VEGFA* and osteogenic genes in response to PHIs in both maintenance and osteogenic induction conditions; after 1 week of treatment, *VEGFA* (**A**) was upregulated with DMOG and to a lower extent with baicalein in both MM and OM. Relative gene expression levels for *RUNX2* (**B**), *ALPL* (**C**), and *COL1A1* (**D**) declined with both DMOG and baicalein. Baicalein revealed a trend for upregulating *BMP2* and *SPP1* (**E**, **F**). Column graphs show observed mean and SD for three independent biological replicates (dots), *p < 0.05

**Fig. 10:**
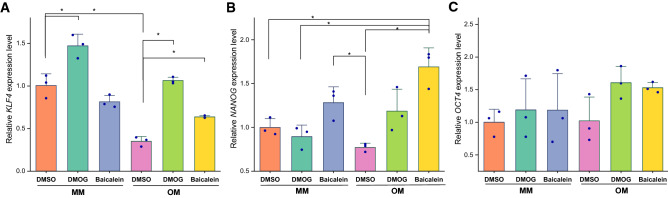
DMOG and baicalein upregulated stemness markers; relative gene expression levels for *KLF4* (**A**), *NANOG* (**B**), and OCT4 (**C**) showed an upregulation trend with both PHIs, which was significantly evident with osteogenic induction for *KLF4* and *NANOG*. Column graphs show observed mean and SD for three independent biological replicates (dots), *p < 0.05

DMOG upregulated *VEGFA* expression more than baicalein (Fig. 9A). Gene expression of *RUNX2* showed largest differences between the means of test conditions and OM + DMSO. However, due to variability in donor response, a significant difference was not detected (Fig. 9B). Tested conditions did not significantly alter *ALPL* gene expression except for OM + DMSO which significantly increased *ALPL* gene expression (Fig. 9C). *COL1A1* gene expression and hydroxyproline content also followed similar trends (Fig. 7B). However, variability in donor responses resulted in only slightly non-significant differences (*p* = 0.07) (Fig. 9D). *BMP2* relative gene expression levels showed the largest difference in means between OM + baicalein and OM + DMSO (Fig. 9E). Gene expression *SPP1* increased in the presence of baicalein, especially in MM (Fig. 9F). Both PHIs upregulated stemness markers *KLF4*, *NANOG*, and *OCT4*, with a significant upregulation trend especially under osteogenic induction (Fig. 10A–C).

## Discussion

In this study we show that tested PHIs can potentially enhance the proangiogenic effects of AT-MSCs in bone engineering strategies via HIF-1α/VEGF pathway. Long-term PHIs treatment, however, can reduce the osteogenic potential of AT-MSCs and rather upregulate stemness markers.

Enhancing vascularization of tissue engineered bone substitutes is crucial for maintaining the cellular oxygenation, nutrition, waste removal, and for recruiting osteoprogenitors and immune cells. Albeit angiogenesis and osteogenesis are different processes; they are nevertheless, critically orchestrated in a series of events during bone development and regeneration process [27].

Both tested PHIs were able to stabilize HIF-1α in a dose-dependent manner, although their stabilization responses were distinct. DMOG and baicalein slowed down proliferation of AT-MSCs in a dose and time dependent manner. Ding and coworkers reported a similar effect of DMOG on AT-MSCs from rats [28]. On the other hand, Marchbank and coworkers showed that DMOG induced a dose-dependent increase in proliferation in human stomach and colonic carcinoma cells with maximal response seen at a concentration of 70 µM. Whereas, at 70–120 μM DMOG inhibited cell proliferation [29]. Baicalein has been reported to have antiproliferative effects on human colorectal cancer cells and hepatocellular carcinoma cell lines [30, 31]. The use of initial concentrations from the short-term cytotoxicity assays for treating AT-MSCs under osteogenic induction suggested a considerable effect of treatment duration on cell viability (data not shown). Such findings required setting up a viability dose–response for AT-MSCs upon treatment with tested drugs for a longer time period (14 days). We emphasized the importance of integrating different physical parameters of the experiment in effective drug concentration calculation, e.g., cell-seeding density, plate well-size, and volume of the media. This was feasible by considering the PHIs concentrations in mole/cell. Expressing drug doses this way improves translatability of experiments between labs as was suggested by Doskey et al. [26].

Hypoxic response effects on cellular proliferation are largely dependent on oxygen levels as well as the duration of hypoxia [32, 33]. In our study, we showed the impact of the hypoxia-mimicking agents’ concentration and duration of treatment on cellular proliferation. Therefore, we focused on developing a methodology to target a concentration-window that achieved the desired proangiogenic signaling while maintaining the cellular proliferation capacity even during prolonged exposure to the tested PHIs. The optimized concentrations for long-term treatment of AT-MSCs were estimated based on the accepted relative viability percentage of 70% [34, 35]. Verification of the suitability of the optimized concentrations also involved cell cycle analysis and apoptosis assay. The results did not show significant cellular apoptosis or necrosis at tested concentrations, however, cell cycle analysis showed a trend towards increased G2/M phase, which does not necessarily imply cell cycle arrest, but rather infer a delay or slow-down in the cell cycle due to the repair mechanisms that block the entry of the cells into mitosis.

HIF-1α/VEGF pathway is critical for coupling angiogenesis and osteogenesis during both skeletal development and postnatal bone regeneration [27, 36, 37]. However, the effects of HIF-1α pathway targeting on osteoblasts and osteoprogenitors are disputed. Previous studies have demonstrated hypoxia to exhibit no or even negative effects on osteogenesis by osteogenically-induced bone marrow-derived mesenchymal stem cells (BMSCs) and osteoblasts [33, 38–41]. On the other hand, other studies have reported that BMSCs undergo osteogenic differentiation in response to hypoxia [42]. Wagegg and coworkers showed that hypoxia promoted osteogenesis of human BMSCs and chemically-induced hypoxia had nearly similar effect when agents were applied once weekly [43]. We see the large heterogeneity in treatment duration and experimental settings behind the contradicting reports.

In our hands, DMOG abolished the effects of osteogenic induction of AT-MSCs, based on gene expression levels and matrix formation. Other research groups have found that treatment with DMOG can stimulate osteogenic and angiogenic differentiation of human BMSCs [44, 45]. The observed difference might be attributed to differences in the cell source, state of differentiation, duration of treatment, or applied effective concentrations. Baicalein attenuated the osteogenic induction on matrix formation, despite overexpressed *BMP2* and *SPP1* genes, and increased levels of osteogenic cytokines such as PDGF-BB, TGF-β2, TGF-β3, and osteopontin. Tested PHIs showed the same effect on osteogenic potential of AT-MSCs even at lower concentrations (Supplementary Fig. 4).

Cobalt chloride (CoCl_2_) is a recognized chemical inducer of hypoxic response in cell culture [46, 47]. Using CoCl_2_ to treat AT-MSCs, in the same experimental protocol of this study, revealed a similar effect of CoCl_2_, like tested PHIs, on cellular proliferation and osteogenic potential of AT-MSCs (supplementary Figs. 5 and 6). Such findings indicate a common effect of the prolonged chemically-induced hypoxia; with tested PHIs and CoCl_2_, on the proliferation and osteogenic differentiation of AT-MSCs.

Interestingly, both PHIs upregulated stemness markers *KLF4*, *NANOG*, *and OCT4*. Such findings are in accordance with Menon and coworkers, who described a similar effect of DMOG on human tendon stem cells [48]. In another setting, we found that such trend held true upon the treatment of AT-MSCs with PHIs for successive passages in regular culturing media. The observed overexpression of *OCT4* and *NANOG* in the higher passages implies a clear effect of the long-term exposure to PHIs, as AT-MSCs seemed to adapt to the hypoxic response by maintaining their stemness as well as proliferative capacity, as previously reported [49]. In accordance with the cell cycle analysis and apoptosis assay results, the tested concentrations of PHIs did not cause cell cycle arrest as evidenced by the repeated passaging of the AT-MSCs under continuous exposure to PHIs (supplementary Fig. 7).

The effects of the tested chemicals on the cell cycle and osteogenic differentiation could also involve a role of the upregulated *OCT4* gene, as revealed by qRT-PCR. *OCT4* has been suggested to promote G1/S transition and reduce differentiation, while also controlling cell cycle regulators to ensure a proper duration of G2 for checking genome integrity and decreasing chromosomal mis-segregation [50, 51]. The prolonged chemically-induced hypoxic response could therefore have helped the AT-MSCs adapt for maintaining their stemness as well as proliferative capacity.

HIF-1α has been proposed having an inhibitory effect on Wnt/β-catenin pathway, this inhibition has been shown to be at least partially due to the HIF-1α-mediated activation of the Wnt antagonist sclerostin (SOST) [52]. Interestingly, we found a similar trend for *SOST* upregulation, especially with DMOG treatment (supplementary Fig. 8), which could be associated with the HIF-1α stabilization effect and suggest a possible mechanism for the attenuated osteogenic response.

In conclusion, our results show that prolonged HIF-1α activation in AT-MSCs inhibited their osteogenic differentiation and directed them towards stemness. Such findings suggest an intimate correlation between stemness and prolonged hypoxic response, potentially simulating the hypoxic niches of MSCs, where they maintain their stemness and self-renewal properties. Despite the reduced osteogenic potential, PHIs-treated AT-MSCs showed enhanced proangiogenic properties, while maintaining their stemness. This can be advantageous for improving AT-MSCs survival and engraftment *in vivo*, and for boosting their regenerative potential which should be explored in future *in vivo* experiments.

## Electronic supplementary material

Below is the link to the electronic supplementary material.

Supplementary material 1 (DOCX 5140 kb)
