# Supplementary material for: Proangiogenic Hypoxia-Mimicking Agents Attenuate Osteogenic Potential of Adipose Stem/Stromal Cells
Source: Tissue Eng Regen Med. 2020 May 24;17(4):477–93. doi: 10.1007/s13770-020-00259-3 (PMC7392999; doi:10.1007/s13770-020-00259-3)
Supplement: Supplementary file 1 — Supplementary material 1 (DOCX 5140 kb) [file 13770_2020_259_MOESM1_ESM.docx]

**Supplementary Figures**

**
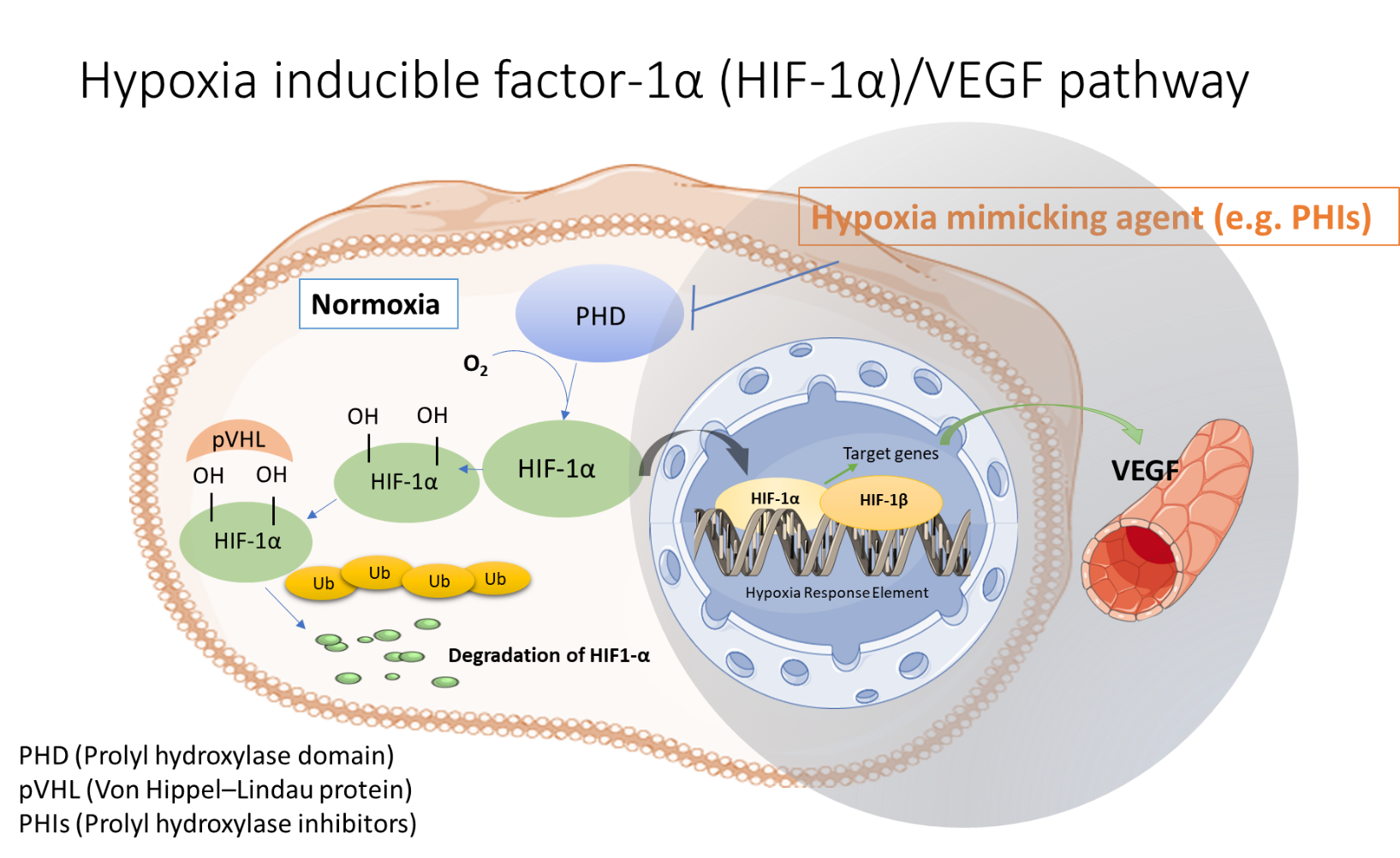
**

**Supplementary Fig. 1:** Graphical illustration for targeting HIF-1α/VEGF pathway by PHIs

**
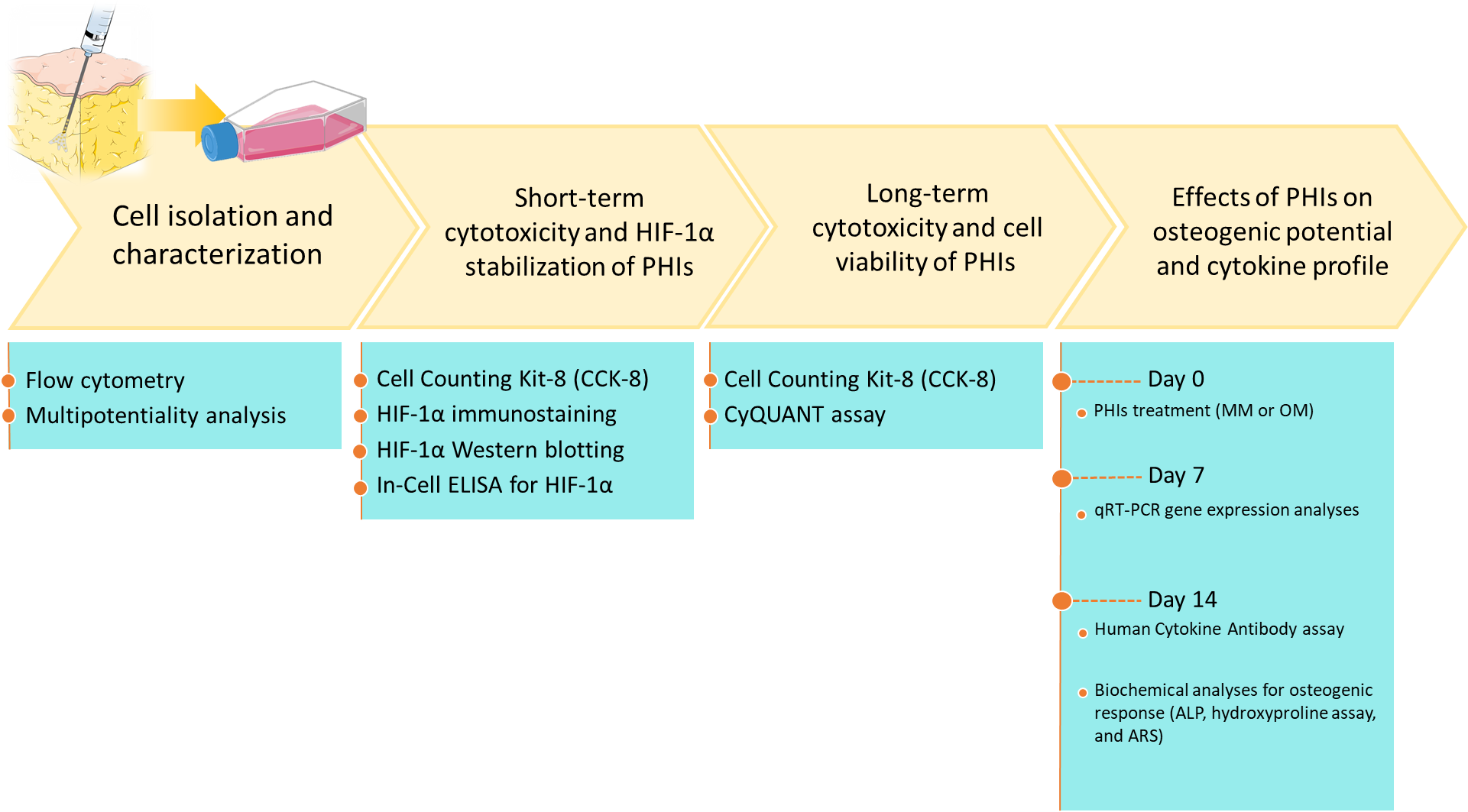
**

**Supplementary Fig. 2:** Graphical illustration for the experimental setup workflow

MM

OM

1 week

2 weeks

3 weeks


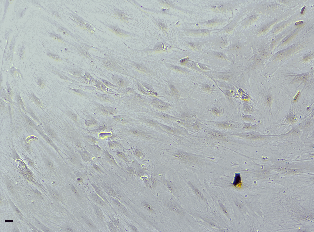

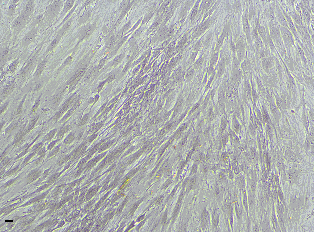

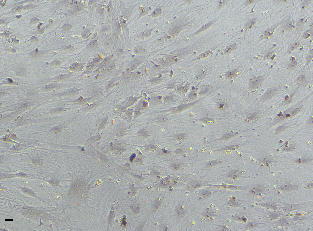

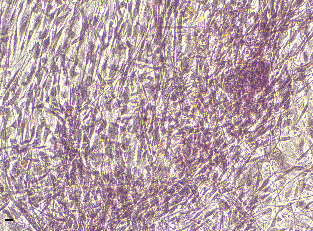

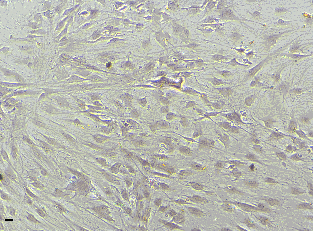

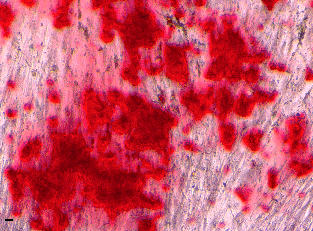


**Supplementary Fig. 3:** shows Alizarin Red S staining for mineralized matrix formation upon osteogenic induction of AT-MSCs. Mineralized matrix was not detected in control conditions (MM), while osteogenically induced cultures (OM) showed gradual formation of a mineralized matrix. The osteogenic response gradually increased, with the earliest mineralized matrix started after 14 days of osteogenic induction up to 21 days. Maintenance medium (MM), osteogenic medium (OM), scale bar 200 μm.


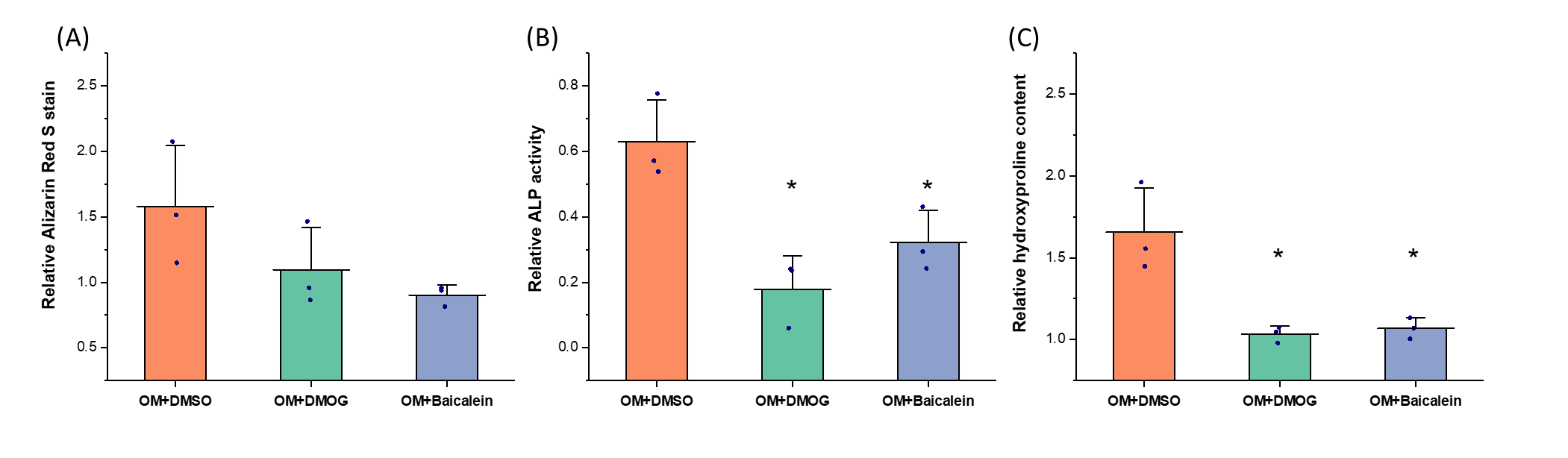


**Supplementary Fig. 4:** Verification of the effects of the tested chemicals at lower concentrations on osteogenic differentiation. As a reference, retested concentrations were estimated based on achieving at least 70 % proliferation of AT-MSCs after 14 days of treatment. Lower concentrations of tested PHIs (i.e., 2.5 pM/cell of DMOG and 0.21 pM/cell of Baicalein) attenuated AT-MSCs osteogenic induction. PHIs reduced the mineralization of extracellular matrix as revealed by ARS assay **(A)**, the alkaline phosphatase activity **(B)**, and hydroxyproline content **(C)**. Column graphs show observed mean and SD for three independent biological replicates (dots), * *p* < 0.05 compared to DMSO control.

**Supplementary Fig. 5:** Results of CCK-8 assay for cellular proliferation of AT-MSCs; treated with different concentrations of CoCl2 for different timepoints, revealed a significant effect for both the concentration and duration of treatment. Column graphs show observed mean and SD for three independent biological replicates (dots), * *p* < 0.05 compared to 0 μM control.


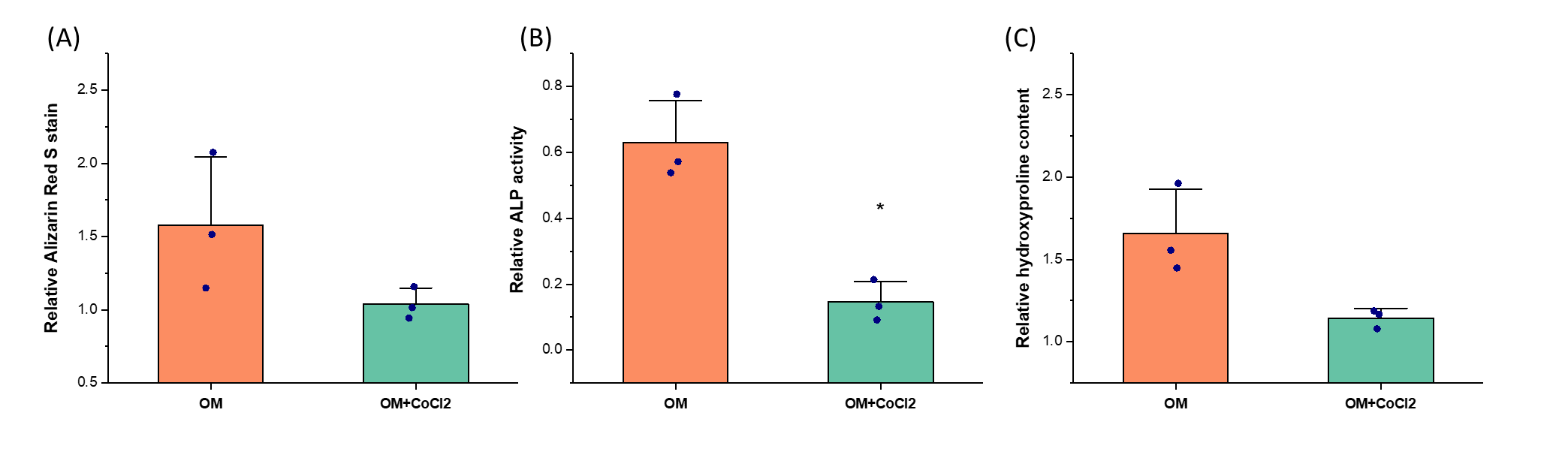


**Supplementary Fig. 6:** CoCl_2_ attenuated AT-MSCs osteogenic induction. CoCl_2_ was used at the concentration which achieved 70 % viability of AT-MSCs after 14 days of treatment. Based on the cellular proliferation assay, it was estimated to be 25 µM equivalent to 1.6 pM/cell. CoCl_2_ reduced the mineralization of extracellular matrix as revealed by ARS assay **(A)**, the alkaline phosphatase activity **(B)**, and hydroxyproline content **(C)**. These findings paralleled the effects of DMOG and baicalein on osteogenic differentiation of AT-MSCs. Column graphs show observed mean and SD for three independent biological replicates (dots), * *p* < 0.05 compared to OM control condition.


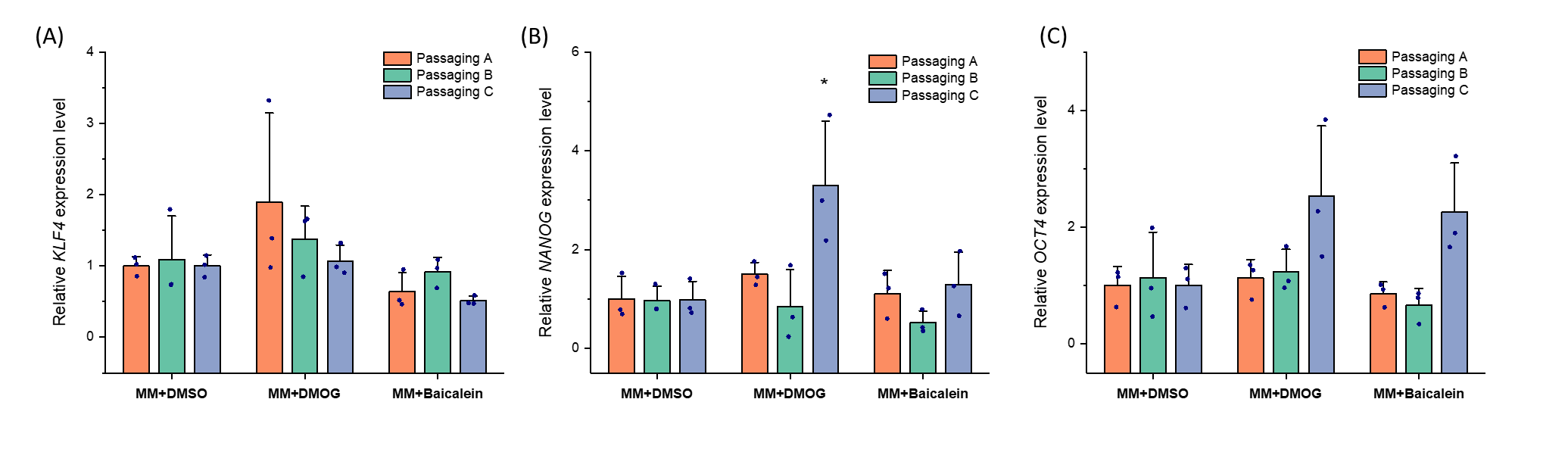


**Supplementary Fig. 7:** Relative gene expression levels for *KLF4* **(A)**, *NANOG* **(B)**, and OCT4 **(C)** upon treating AT-MSCs with tested PHIs with repeated passaging. Passaging A refers to first assessed passages (P4 of donors 1 & 3 and P5 for donor 2), ongoing analysis of subsequent passages was carried out till passaging C (i.e., P6 for donors 1 & 3 and P7 for donor 2). The results showed an upregulation trend in *OCT4* and *NANOG* with both PHIs in the latest passage. Column graphs show observed mean and SD for three independent biological replicates (dots), * *p* < 0.05.

**
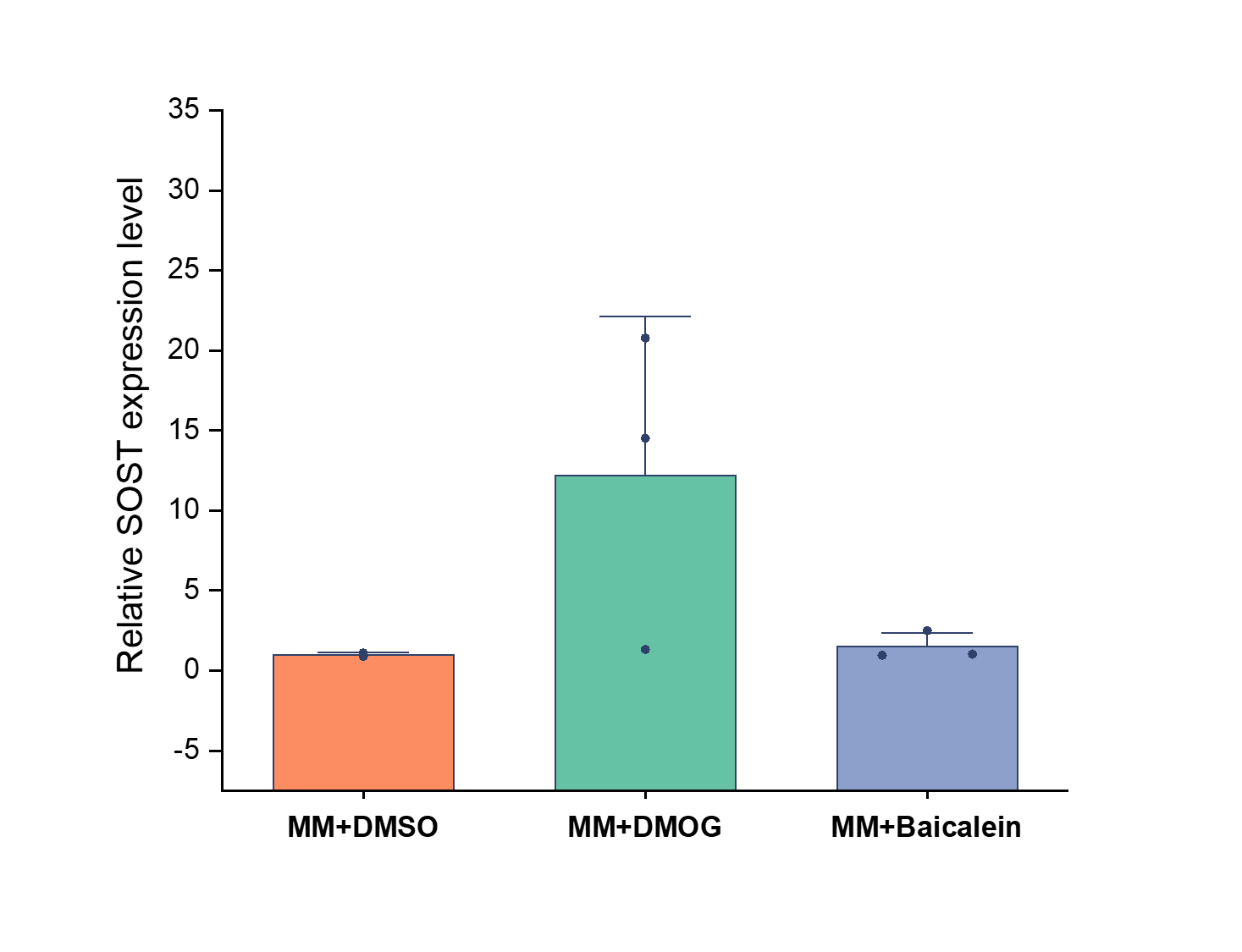
**

**Supplementary Fig. 8:** shows qRT-PCR relative sclerostin gene expression (*SOST*, TaqMan assay ID Hs00228830_m1), which was upregulated especially with DMOG treatment. Column graphs show observed mean and SD for three independent biological replicates (dots).
